# Women Living with HIV over Age of 65: Cervical Cancer Screening in a Unique and Growing Population

**DOI:** 10.1155/2017/2105061

**Published:** 2017-09-17

**Authors:** Alexandra Aserlind, Karla Maguire, Lunthita Duthely, Stefan Wennin, JoNell Potter

**Affiliations:** University of Miami Miller School of Medicine, Department of Obstetrics & Gynecology, Miami, FL, USA

## Abstract

**Objective:**

Women living with HIV are at increased risk of human papillomavirus (HPV) infection, which can lead to cervical cancer. New guidelines recommend indefinite screening. The objective of this study is to describe cervical cancer screening practices and colposcopy results in a cohort of women living with HIV over age of 65 who were followed before the new guidelines. Comorbidities, sexually transmitted infections (STIs), and other risk factors were evaluated.

**Methods:**

We conducted a retrospective chart review on 75 women aged 65 or older living with HIV with at least one Pap smear.

**Results:**

The mean age of the cohort was 66.5 and at HIV diagnosis was 56. The majority of women were immunocompetent. 80% had serial Pap smears. Of these, 86% of 238 were negative or ASCUS. No women progressed to HSIL. 92% of colposcopies had negative or CIN I results. Three women were treated successfully for high-grade dysplasia. More than half of women had other STIs. 72% were screened for HPV; 50% were positive.

**Conclusion:**

The majority of women had negative and low-grade Pap smears. Questions remain regarding the utility of continued Pap screening and the added value of HPV testing in this unique population of older women living with HIV.

## 1. Introduction

 The number of older people living with Human Immunodeficiency Virus (HIV) in the United States is increasing [1]. From 2008 to 2011, the prevalence of HIV infection in people aged 65 and older grew by 41%, making the rate of increase higher than in any other age group [2]. This is due to increased rates of HIV transmission in the elderly and the prolongation of life with the advent of combined antiretroviral therapy three decades ago [3]. The population of postmenopausal women living with HIV (WLWH) has grown steadily. The median survival time after diagnosis in women over 50 years of age increased by 11 years from 1996 to 2014, reaching an average of 22.8 years.

With the prevalence of elderly WLWH expected to double over the next 10 years, there will be an increasing need for expert gynecological care for these women [4]. WLWH are at an increased risk of coinfection with high-risk human papillomavirus (HPV) and have higher rates of developing precursor lesions that may potentially lead to cervical cancer. The rates of high-grade cervical dysplasia and HPV coinfection are directly related to HIV viral load and inversely related to CD4 count. Multiple studies suggest a decrease progression of neoplasia with use of antiretroviral therapy [5, 6]. As CD4 counts drop below 200 cells/*μ*L, there is a 2-fold increase in the incidence and prevalence of cervical preinvasive lesions such as cervical intraepithelial neoplasia (CIN) and low-grade squamous intraepithelial lesion (LSIL), when compared to those with CD4 counts > 500 cells/*μ*L [7, 8].

Cervical screening and management guidelines for this aging population continue to evolve. Previous guidelines recommended cervical cytology twice in the first year after HIV diagnosis and annually thereafter, with no guidance on stopping [6]. The latest cervical screening guidelines for WLWH over 65 years of age, published in 2016, recommend cervical cancer screening to be continued for the duration of a woman's life [9]. This is in contrast to guidelines in place for women without HIV, which support cessation of screening after 65 years of age, subsequent to three consecutive negative cytology results. Alternately, women not living with HIV can suspend cervical cancer screening with two consecutive negative HPV cotest results within the past 10 years, with the most recent test having taken place within the past 5 years [10]. Guidelines also recommend colposcopy for WLWH with ASCUS who test positive for HPV, low-grade squamous epithelial lesion (LSIL), ASCUS-cannot rule out high grade (ASC-H), or high-grade squamous epithelial lesion (HSIL) [6, 9]. If cytology yields ASCUS but HPV testing is not available, practitioners are instructed to repeat cytology in 6–12 months; if ASCUS results again, the patient should undergo colposcopy [9].

In women without HIV, infections with HPV are mostly transient; however, persistent infection 1-2 years after initial infection is a strong predictor of subsequent risk of cervical cancer [9, 11]. Although persistent HPV infections are more commonly seen in women over 65, cervical cancer occurs a median of 15–25 years after HPV infection, calling into question the utility of screening in elderly women [9]. Women in the general population aged 65 and older constitute 19.6% of the incident cases of cervical cancer in the United States, despite the fact that they make up only 14.1% of this country's female population, suggesting age and related comorbidities are risk factors themselves [12]. Lastly, epithelial atrophy in elderly women may lead to false-positive cytology results and subsequent unnecessary Pap smears and pain [9, 13].

A literature search was performed using PubMed and MEDLINE databases, with search terms “HIV-positive,” “elderly,” “over 65,” “cervical screening,” and “pap smears.” No studies were found that describe serial Pap smear screening in this unique population. This study aims to report on Pap smear screening among a group of WLWH over 65 years of age, who were followed before the new guidelines were issued in 2016. The primary objective of this descriptive study was to report on serial cervical cancer screening and colposcopy results in this unique cohort. Secondary objectives included a description of comorbidities and risk factors in this unique and important aging population of WLWH.

## 2. Materials and Methods

A retrospective chart review was conducted on 75 WLWH over the age of 65 seen during 2005 to 2015 at an urban, public hospital clinic in Miami, Florida. This study was approved by the Institutional Review Board at the University of Miami. Data source consisted of a hospital electronic medical record (EMR) and the CAREW are database dedicated to WLWH receiving HIV and gynecologic specialty care. All women confirmed positive for HIV, aged 65 years or older, with one or more Pap smear results after the age of 65, were included in the study. Women younger than 65 and women over 65 with inadequate cervical screening documentation were excluded.

Information collected on the WLWH included demographics, age of HIV diagnosis, antiretroviral history, CD4 cell counts, viral load, cervical cytology, colposcopy, and any treatment results. Presence of comorbidities (diabetes, hypertension, cardiovascular disease, dyslipidemia, and mental health), laboratory-confirmed sexually transmitted infection (STI) results (gonorrhea, chlamydia, herpes, syphilis, trichomoniasis, and Hepatitis B), and risk factors (substance abuse and smoking) were collected. Pap smear results were recorded according to the Bethesda classification system [12]. All of the extracted data were entered into an Excel spreadsheet that identified study subjects only by ID number and stored in a password-protected computer.

Descriptive statistics were calculated for study sample characteristics, Pap smear screening tests, colposcopy, and sexually transmitted infection testing including frequencies, univariate percentages, proportions, means, and standard deviations. Data was analyzed using IBM SPSS Statistics for Windows, Version 22.0 (Armonk, NY).

## 3. Results

A total of 75 records were included in the analyses. The average age of the WLWH over 65 was 66.5 (SD 3). A majority of the study population were Black non-Hispanic (69%). The mean age at HIV diagnosis was 56 (SD 7) years. 80% of women had received an AIDS diagnosis, and the vast majority was on antiretroviral therapy at the time of the first Pap smear after age of 65. Most women were immunocompetent; 92% of women had CD4 counts greater than 200 cells/mm^3^; and 53% had CD4 counts greater than 500 cells/mm^3^. 84% of women had a viral load that was undetectable (defined as less than 50 copies/mL). The majority of the women (80%) were below the federal poverty level. These values are presented in Table 1. Many of these women had other medical comorbidities, most notably hypertension (80%). Approximately 20% of the women admitted to smoking cigarettes. Rates of medical and psychiatric comorbidities, STI, and HPV screening and social risk factors in this group are seen in Table 2.

All WLWH included in the study had at least one Pap smear after turning 65 years of age. A total of 238 Pap smears were collected among the 75 women in this study group, of which 86% were negative or ASCUS and 13% were LSIL. Only three Pap smears (1%) yielded a high-grade (HSIL) result. The three HSIL results occurred with one patient (Table 3).

Forty-four women had three or more Pap smears performed (defined as serial Pap smear screening) collected at least one year apart (see Table 4). These serial Pap smear results were then tabulated, by comparing the first Pap smear to the last Pap smear. Of the 44 women, six (14%) progressed to LSIL. None of the women with serial Pap smears progressed to HSIL. The majority of women (34, 77%) with a negative or ASCUS first Pap did not progress beyond ASCUS. A total of 21 (91%) of the 23 women whose first Pap smear was negative did not progress beyond ASCUS, and 13 (81%) of the 16 women whose first Pap smear was ASCUS did not progress beyond ASCUS (Table 4).

Thirty (40%) of women had at least one colposcopy, with a total of 49. When Pap smears were matched with the respective colposcopy result the majority (92%) returned negative or CIN 1. Two of the abnormal colposcopy results were CIN 2, and one result was CIN 3. One of the women with CIN 2 had a subsequent loop electrocautery excision procedure (LEEP), and follow-up Pap smear conducted eight months later resulted in LSIL. The second woman with CIN 2 underwent a cold knife cone procedure, which returned negative for dysplasia. The woman with CIN 3 underwent a LEEP with follow-up Pap smear testing showing ASCUS (Table 5).

Pap smears were considered and linked to the colposcopy if the colposcopy was performed within 6 months. Not every ASCUS, LSIL, or HSIL Pap was matched to a colposcopy result. Of the 66 instances colposcopy was indicated; 40 (61%) were done. A total of 26 (39%) recommended colposcopies were not done within 6 months. This was due to either no-show to colposcopy appointments, lost to follow-up, or the clinical decision to repeat ASCUS Pap smears in 6 months.

## 4. Discussion

In this population of WLWH over 65 years old, the mean age of HIV diagnosis was 56. The vast majority of these women had negative or low-grade Pap smear results, and only one patient had Pap smears with high-grade results. Among the women with serial Pap smear screening (3 or more) the majority did not progress beyond ASCUS. No one in this study progressed to HSIL. Of women who underwent colposcopy, only three required treatment of their cervical dysplasia. Over half of the women had an STI during the study period.

The age at HIV diagnosis is consistent with national trends. Since the majority of the women were over the age of 55 at the time of HIV diagnosis and 57% had an STI during the study period, a comprehensive sexual history should be elicited in older women in addition to routine screening for HIV and STIs, including HPV. Only 72% of WLWH in our cohort were tested for HPV, exposing an area where improved screening can be implemented.

This study does have limitations. Primarily, due to its retrospective chart review design, data is restricted to what is recorded in the medical record. Secondly, 48% of women who required colposcopy based on Pap smear results did not have one for various reasons. Lastly, in our institution HPV testing was not routine, so HPV status could not be reviewed as an adjunct to Pap testing in this population. When HPV testing was available, HPV typing was not always carried out.

The strength of this research is that, to our knowledge, this is the only study focusing on serial Pap smears in WLWH over age of 65, which is a growing population. The utility of continued indefinite cervical cancer screening in a population with many diverse healthcare needs should be further studied and addressed. Future research that includes HPV testing in conjunction with Pap smears could better determine if continued cervical cytology screening is warranted among older women WLWH. Also, studies that compare women over 65 living with HIV to women in the same age group who are HIV negative would facilitate a better understanding of dysplasia in this population.

## Figures and Tables

**Table 1:** Demographic characteristics of HIV+ women > 65 years old.

Characteristic	*n* = 75 *N* (%)
Age: *mean (*±*SD)*	66.5 (±3)

Race/ethnicity	
Black non-Hispanic	52 (69)
Hispanic	22 (29)
White non-Hispanic	1 (1)

Age at HIV diagnosis: *mean (*±*SD)*	56 (±7)

CDC-defined AIDS	60 (79)

HIV transmission risk factor	
Heterosexual contact	72 (96)
IV drug use	2 (3)
Blood products	1 (1)

Taking antiretroviral medication	70 (92)

CD4 count	
<200 cells/mm^3^	7 (9)
200–500 cells/mm^3^	28 (37)
>500 cells/mm^3^	40 (53)

Nondetectable viral load *(<50 copies/mL)*	63 (84)

Primary insurance	
Medicare/Medicaid	60 (80)
Uninsured	15 (20)

At or below federal poverty level	60 (80)

**Table 2:** Comorbidities and risk factors in HIV+ women > 65 years old.

	*n* = 75 *N* (%)
*Medical*	
Hypertension	60 (80)
Dyslipidemia	31 (41)
Diabetes	21 (28)
Cardiovascular/MI	7 (9)

*Gynecological*	
Sexually transmitted infection (STI)	
*(one or more)*	43 (57)
STI stratification	(*n* = 43)
Hepatitis B	30 (70)
Syphilis	23 (54)
Herpes simplex virus	5 (12)
Neisseria gonorrhea	0 (0)
Chlamydia trachomatis	0 (0)
Trichomoniasis	0 (0)

HPV testing	(*n* = 54)
Positive	27 (50)
Negative	27 (50)

*Psychiatric/cognitive*	
Mental illness	11 (15)
Dementia	4 (5)

*Social factors*	
Cigarettes	17 (23)
Alcohol abuse	11 (15)
Illicit drugs	7 (9)

**Table 3:** Results of all Pap smears results for HIV+ women > 65 years old.

Pap smear rsesults	*n* = 238 *N* (%)
Negative	118 (50)
ASCUS	85 (36)
LSIL	32 (13)
HSIL	3 (1)

**Table 4:** Pap smear progression: first Pap smear to last Pap smear, for HIV+ women > 65, with 3 or more Pap smear results (*n* = 44).

PAP smear (first result)	Pap smear result (last result)				
	Negative	ASCUS	LSIL	HSIL	Total
Negative	13 (56%)	8 (35%)	2 (9%)	—	23
ASCUS	5 (31%)	8 (50%)	3 (19%)	—	16
LSIL	1 (25%)	2 (50%)	1 (25%)	—	4
HSIL	—	1 (100%)	—	—	1

Total	19	19	6	0	44

**Table 5:** Pap smears with matched colposcopy results within 6 months for HIV+ women > 65 (*n* = 40).

PAP smear	Colposcopy results				
	Negative	CIN 1	CIN 2	CIN 3	Total
ASCUS	13	8	1	0	22
LSIL	5	10	1	0	16
ASC-H/HSIL	0	1	0	1	2

Total	18	19	2	1	40

## Additional Points

*Precis*. Most older women living with HIV had negative/low-grade cytology; however further research is needed to demonstrate the value of screening.
